# Diagnosis of antigenic markers of acute toxoplasmosis by IgG avidity immunoblotting

**DOI:** 10.1051/parasite/2013017

**Published:** 2013-05-22

**Authors:** Siamak Ali-Heydari, Hossien Keshavarz, Saeedeh Shojaee, Mehdi Mohebali

**Affiliations:** School of Public Health, Tehran University of Medical Sciences Tehran Iran

**Keywords:** toxoplasmosis, avidity immunoblotting

## Abstract

To perform IgG avidity immunoblotting assay for detection of acute toxoplasmosis, 100 serum samples were collected from Tehran, Iran. The presence of *Toxoplasma*-specific IgG and IgM antibodies were checked by commercial Trinity kit. Samples were categorized in acute and chronic phases of *Toxoplasma gondii* infection according to IgG avidity ELISA. IgG avidity immunoblotting was performed, and antigenic bands with molecular weights of 22, 25, 28, 30, 32, 42, 44, 49, 55, 60, 66, 69, 88, 106, 130 and 157 kDa were recognized as low avidity markers. The most prevalent antigen for low avidity was p22. It is concluded that IgG avidity immunoblotting could distinguish acute and chronic phases of *T. gondii* infection.

## Abstract (fr)

Pour exécuter un essai d’immunoréplique d’avidité des IgG pour la détection de la toxoplasmose aigue, cent échantillons de sérums ont été collectés à Téhéran, Iran. La présence d’anticorps IgG et IgM spécifiques de *Toxoplasma* a été vérifiée par le kit commercial Trinity. Les échantillons ont été caractérisés comme phases chronique ou aigue d’infection par *Toxoplasma gondii* en fonction d’avidité ELISA des IgG. Une immunoréplique d’avidité des IgG a été effectuée, et les bandes antigéniques de poids moléculaires de 22, 25, 28, 30, 44, 49, 55, 60, 66, 69, 88, 106, 130 et 157 kDa ont été reconnues comme des marqueurs de basse avidité. L’antigène le plus prévalent pour la basse avidité était p22. On conclut que l’immunoréplique d’avidité des IgG pourrait distinguer les phases chroniques ou aigue d’infection par *T. gondii*.

## Introduction

*Toxoplasma gondii* is an obligate intracellular parasite that invades the host cell, replicates and finally leads to the lysis of the cell [3]. *T. gondii* is associated with congenital infection and it can cause encephalitis, or systemic infection in immunocompromised patients [14]. It is important to know whether the infection is recently acquired or is chronic. Differentiation between acute and chronic infection has a dramatic impact, especially for the developing fetus [2]. Maternal infection diagnosis is based on serological methods with detection of specific IgG and IgM antibodies [7]. In some cases, the IgM antibody remains long-lasting. Thus the presence of IgM or IgG antibodies cannot define whether the infection is acute or chronic [11]. The avidity of *T. gondii* antigen to specific antibodies can change during the courses of infection. With this regard, in the acute stages, the avidity is low and increases with the duration of infection [5]. In the IgG avidity ELISA test, protein denaturing agents, such as urea, are used to dissociate the antigen- antibody complex. The result is determined by using the ratio of optical density of urea-treated and untreated wells. In some individuals, low avidity may persist for one year. It is difficult to interpret the results in the IgG avidity test with the presence of IgM antibody, so low avidity does not mean recent infection in all the patients [13]. In some studies, avidity immunoblotting has been used for detection of antigenic markers of acute or chronic toxoplasmosis [1, 10], in which different profiles of antigenic markers could be detected with or without washing with urea.

To distinguish the acute *T. gondii* infection, this study was performed to detect immunological markers by IgG avidity immunoblotting.

## Materials and methods

This study was approved by the Ethical Committee of Tehran University of Medical Science. One hundred serum samples were collected from different laboratories in Tehran, Iran. These referred sera were positive for *T. gondii*-specific IgG and IgM antibodies according to indirect immunofluorescent antibody test. In our laboratory the presence of IgM and IgG antibodies were confirmed by IgG and IgM Trinity kit according to the manufacturer recommendations (Trinity, USA). IgG avidity ELISA was performed and the sera were divided in acute and chronic groups according to the avidity ELISA results.

### IgG avidity ELISA

Tachyzoites of *T. gondii* RH strain were inoculated intraperitoneally in Balb/c mice. All mice were treated in accordance to the principles of laboratory animal care (Laboratory Animal Care Facility, Standard Operating Procedures, Western Illinois University). Male mice were housed in rooms with temperature of 20–23 °C, with constant humidity (40–50%), a 12 h light/dark cycle, and were fed with the standard diet and water.

The tachyzoites were collected by performing mice peritoneal washing with sterile normal saline (pH 7.2). The exudates were centrifuged at 3,000g, washed, sonicated (15 × 10 s) and finally centrifuged at 4 °C and 12,000g for 1 h. The supernatants were collected and the protein amounts were measured by the Bradford method.

The 96 well micro-titer plates (Nunc, Denmark) were coated with 5 μg/mL of soluble antigens in carbonate-bicarbonate buffer (pH 9.6) overnight at 4 °C. Plates were washed for three times with PBST (PBS, 0.05% tween20), then sera diluted in PBST (1:200) were added in duplicate rows (row A and row B). After incubation for 1 h at 37 °C, row B was washed three times with PBST, and row A was washed three times with PBST containing 6 M urea and a fourth time with PBST. Then antihuman IgG conjugated with horseradish peroxidase (Dako, Denmark) at the dilution of 1/1,000 in PBST was added and incubated for 1 h, followed by addition of the ortho-phenylendiamidine (OPD), (Merck, Germany) substrate. The reaction was stopped and the absorbance (Abs) was read by an automated ELISA reader (BIOTEC, LX800, USA) at 492 nm. Avidity index (AI; %) was calculated as the result of Abs of wells washed with PBST containing urea (U+), divided by the Abs of wells washed with PBST (U−), and multiplied with 100, based on the formula; AI = Abs(U+)/Abs(U−) × 100.

### IgG avidity immunoblotting

The soluble antigens of *T. gondii* RH strain were prepared as mentioned for avidity ELISA. The soluble antigens (10 μg/well), and molecular weight markers (chromatein prestained protein ladder, Vivantis) were run on 12% polyacrylamide electrophoresis gel at 150 V for 3 h. The gel was transferred onto nitrocellulose paper (Porablot, Germany) under a current of 50 mA overnight. The paper was cut, the marker separated, and the strips of paper blocked with 3% skimmed milk (Merck, Germany) in PBST for 2 h. Each diluted sera (1:50) in PBST was added in duplicate on nitrocellulose strips. After overnight incubation, one strip was washed with PBST and the other washed with PBST containing 6 M urea for 5 min three times. The fourth washing was done with PBST for all the strips. Then, the antihuman IgG conjugated with horseradish peroxidase (HRP) (Dako, Denmark) at the dilution of 1/500 in PBST was added. After incubation for 1 h and washing for three times the diaminobenzidine (DAB) (Sigma, USA) substrate was added. The reaction was stopped by rinsing of the strips with distilled water. To determine the specificity of avidity immunoblotting, 10 negative sera for *Toxoplasma-*specific IgG and IgM antibodies were tested too.

## Results

According to the Trinity kit from the 100 *Toxoplasma*-specific IgG positive sera tested, 23 samples had *Toxoplasma*-specific IgM antibody too. According to the results of IgG avidity ELISA, from the 100 IgG positive sera, 16 samples had AI ≤ 50, so were placed in acute phase of infection and 84 samples had AI > 50 and were placed in chronic phase of infection.

**Figure 1.**
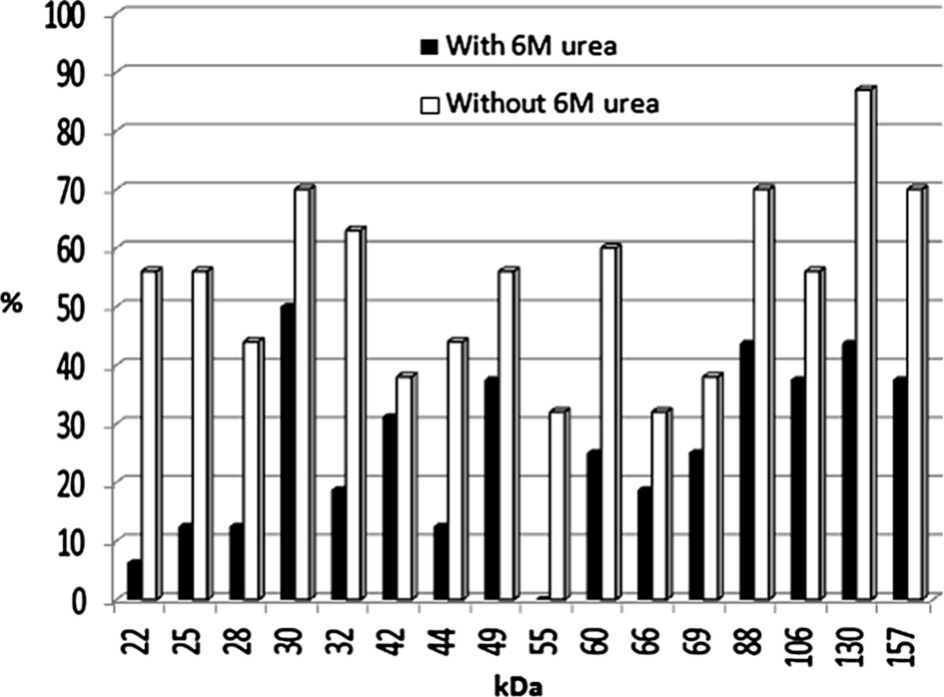
Frequency of antigenic bands after washing without or with 6 M urea in acute phase of *T. gondii* infection by avidity immunoblotting.

**Figure 2.**
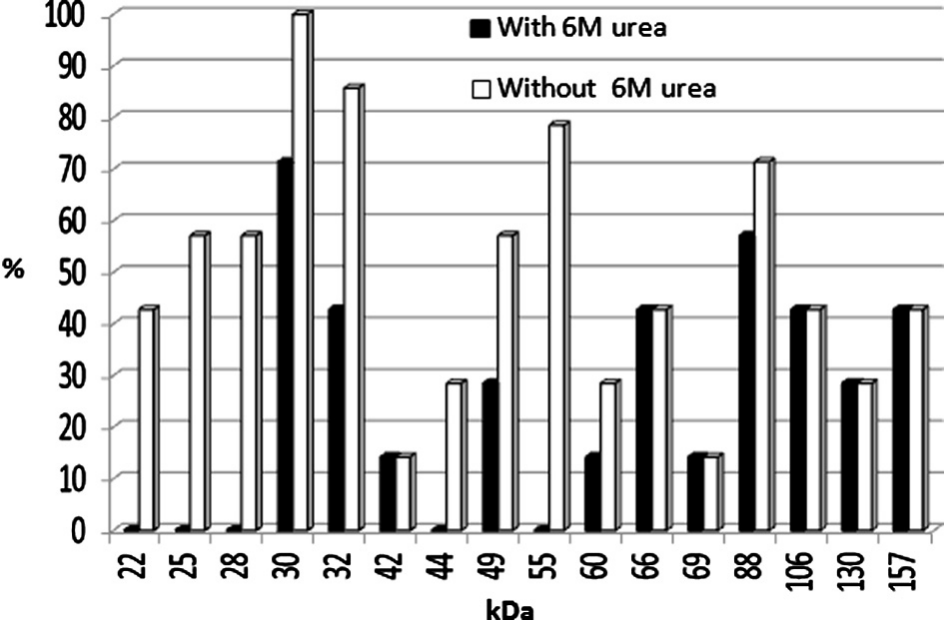
Frequency of antigenic bands after washing without or with 6 M urea in *T. gondii* IgM positive samples with AI > 50 by avidity immunoblotting.

**Figure 3.**
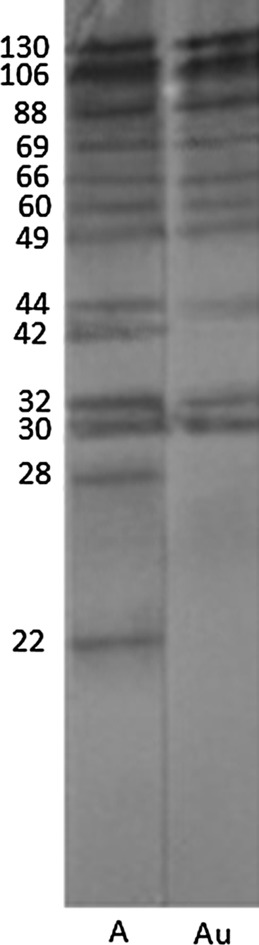
Antigenic bands after washing without or with 6 M urea in *T. gondii* infection by avidity immunoblotting. A: acute phase washed without urea, Au: acute phase washed with urea.

The acute group with AI ≤ 50 had shown different antigenic profiles after washing with PBST, containing 6 M urea by IgG avidity immunoblotting. The differences were seen in 22, 25, 28, 30, 32, 42, 44, 49, 55, 60, 66, 69, 88, 106, 130 and 157 kDa antigenic bands. The most prevalent difference was seen with the 22 kDa band after washing with PBST containing urea (Figures 1 and 3). In the group with AI > 50 the antigenic profile was similar in 77 samples. Seven samples had different antigenic profiles after washing with PBS containing 6 M urea (Figure 2). These seven samples had IgM antibody according to Trinity kit.

No positive reaction was seen in negative sera by avidity immunoblotting.

## Discussion

In order to make a reliable decision about the treatment of *T. gondii* infection, it should be known whether the infection is in the acute or chronic stage. Thus supplementary methods, which can differentiate between the early and late stage of toxoplasmosis are needed [8]. IgM antibodies are detectable for one year or more, following the acute phase of infection in some individuals, so the presence of IgM antibodies is not always an indication of a recent infection [10]. In recent years major efforts have been made improving the ability to diagnose recently acquired infection in the pregnant women and congenital infection in the fetus and newborn. Early detection of anti-*T. gondii* antibody avidity is a valuable and useful tool for identifying positive IgM, particularly in pregnant women. According to the IgG avidity test, high avidity means that the infection has occurred within the 4–5 months before [4, 11–13].

Interpretation of results of IgG avidity test in presence of IgM antibody is critical because the low avidity may persist up to one year and here, it does not mean the recently acquired infection [9]. By using an immunoblotting method, antigenic markers of acute and chronic phases of *T. gondii* infection could be characterized [10].

The present study was performed to determine specific antigenic bands in acute and chronic phases of toxoplasmosis. Serum samples were tested for the presence of *T. gondii* IgG and IgM antibodies by a commercial ELISA kit. Then IgG avidity ELISA was performed and samples were divided into two groups: acute group with AI ≤ 50 and chronic with AI > 50. After performing IgG avidity immunoblotting, all the samples in acute phase had different antigenic profiles when washed with buffer containing 6 M urea, in comparison to washing with buffer alone.

In this study, antigenic bands with molecular weights of 22, 25, 28, 30, 32, 42, 44, 49, 55, 60, 66, 69, 88, 106, 130 and 157 kDa were recognized as low avidity markers. The p22, which probably corresponds to SAG2, was present in about 56% of sera that disappeared in about 50% of them after washing with 6 M urea, so it seems that the p22 could be a good marker of acute infection in avidity immunoblotting. Araujo and Ferreira (2008) [1] performed IgG avidity immunoblotting against excreted/secreted antigens of *T. gondii* and reported that the 30 kDa antigen was a good marker for acute toxoplasmosis in avidity immunoblotting. In 2011, Habib *et al*. [6] claimed that the 30 kDa antigen was reactive with almost all sera either in acute or latent phases of infection with high avidity IgG in mice model. They found that, using whole tachyzoite extract in IgG avidity immunoblotting, the bands of 10 and 39 kDa could discriminate between recent and latent stages of *T. gondii* infection in mice. In this study the p30 was washed with PBS containing urea in only 20% of sera with acute toxoplasmosis. Marcolino *et al*. (2000) [10] observed that bands p16, p32, p38, p40, p43, p54, p60 and p97 were more frequently recognized by low avidity IgG in recent infection and by high avidity in chronic toxoplasmosis. From these antigenic bands, the p38 was found an optimal antigenic marker of low avidity for recent *T. gondii* infection due to a significant decrease of its frequency after washing with urea.

In this study all the samples in acute phase of *T. gondii* infection showed differences in antigenic bands after washing with urea in comparison to washing without urea. Some IgM positive samples that were checked by commercial ELISA kit had IgG avidity index more than 50 (AI > 50). It was interesting that these mentioned sera had different antigenic profiles in avidity immunoblotting when washed with urea, in comparison with washing without urea. These sera divided in chronic group according to avidity ELISA, but they had IgM antibody against *T. gondii* infection, and they also showed different antigenic profiles in avidity immunoblotting, so the results of avidity immunoblotting and Trinity kit were correlated with each other.

The present study showed that IgG immunoblotting avidity can distinguish acute from chronic phases of *T. gondii* infection defined on the basis of IgG ELISA avidity.
